# Biogenic Fabrication of Iron/Iron Oxide Nanoparticles and Their Application

**DOI:** 10.1186/s11671-016-1714-0

**Published:** 2016-11-11

**Authors:** Khwaja Salahuddin Siddiqi, Aziz ur Rahman, Tajuddin, Azamal Husen

**Affiliations:** 1 Department of Chemistry, Aligarh Muslim University, Aligarh, 202002 Uttar Pradesh India; 2 Department of Saidla (Unani Pharmacy), Aligarh Muslim University, Aligarh, 202002 Uttar Pradesh India; 3 Department of Biology, College of Natural and Computational Sciences, University of Gondar, P.O. Box #196, Gondar, Ethiopia

**Keywords:** Iron, Iron oxide nanoparticles, Biosynthesis, Drug delivery system, Cytotoxicity

## Abstract

Enshrined in this review are the biogenic fabrication and applications of coated and uncoated iron and iron oxide nanoparticles. Depending on their magnetic properties, they have been used in the treatment of cancer, drug delivery system, MRI, and catalysis and removal of pesticides from potable water. The polymer-coated iron and iron oxide nanoparticles are made biocompatible, and their slow release makes them more effective and lasting. Their cytotoxicity against microbes under aerobic/anaerobic conditions has also been discussed. The magnetic moment of superparamagnetic iron oxide nanoparticles changes with their interaction with biomolecules as a consequence of which their size decreases. Their biological efficacy has been found to be dependent on the shape, size, and concentration of these nanoparticles.

## Review

### Introduction

Many physical and chemical methods have been developed for the fabrication of nanoparticles. However, the chemicals used in these procedures leave toxic residues and pollute the environment. Therefore, biogenic synthesis of nanoparticles using fungi, bacteria, actinomycetes, algae, and higher plants have emerged as potential nanofactories which are cost effective and environment friendly [1–8]. The metal and metal oxide nanoparticles are widely used in agriculture, drug delivery, cosmetics, photonic crystals, analysis, food, coatings, paints, catalysis, and material science [1, 9–12].

At present, the application of iron oxide nanoparticles in medical and other sectors appears to be increasing fast [1, 13–17]. Antibacterial activity of *Argemone mexicana* treated with iron oxide nanoparticles against *Proteus mirabilis* and *Escherichia coli* has been reported [18]. Iron nanoparticles fabricated using five different plants (*Lawsonia inermis*, *Gardenia jasminoides*, *Azadirachta indica*, *Camellia sinensis* leaf extract, and *Cinnamon zeylanicum* bark extract) were found to be toxic to many bacterial strains [19].

Table 1 summarizes the physical parameters of iron and iron oxide nanoparticles. Biogenic fabrication of the iron and iron oxide nanoparticles using extracts of different parts of a variety of plants such as *Euphorbia milli*, *Tridax procumbense*, *Tinospora cordifolia*, *Datura innoxia*, *Calotropis procera*, and *Cymbopogon citratus* have also been reported [44]. Magnetic iron oxide nanoparticles have great potential as a drug carrier and MRI agent and in tissue repair and in the treatment of tumor [45]. They are also used as pigments in paints and ceramics and as catalysts for the manufacture of ammonia by Haber’s process and oxidation of alcohols to aldehydes [46–48] and other chemicals. The toxicity of iron oxide nanoparticles can be employed in inhibiting the growth of bacteria, fungi, and other pathogens [49].

**Table 1:** Size and morphology of iron and iron oxide nanoparticles fabricated from plant system

Reference	Plant	Morphology	Size (nm)
[20]	Green tea	Spherical	70–80
[21]	Green tea	Spherical	5–15
[22]	Tea powder	Differs according to the quantity of tea extract	–
[23]	Green tea	Irregular clusters	40–60
[24]	Green tea, oolong tea, and black tea	Irregular spherical	20–40
[25]	Oolong tea	Spherical	40–50
[26]	Sorghum bran	Spherical	40–50
[27]	Eucalyptus	Spherical	50–80
[28]	Eucalyptus	Cubic	40–60
[29]	Eucalyptus	Spherical	20–80
[30]	Pomegranate	–	100–200
[31]	Plantain peel	Spherical	Less than 50
[32]	Banana peel	–	10–25
[33]	Tangerine peel	Spherical	50
[34]	*Dodonaea viscosa*	Spherical	50–60
[35]	*Tridax procumbens*	Irregular spheres	80–100
[36]	Grape seed proanthocyanidin (GSP)	–	Around 30
[37]	Pomegranate, mulberry, and cherry	–	10–30
[38]	Vine leaves, black tea leaves, and grape marc	–	15–45
[39]	*Terminalia chebula*	Chain-like	Less than 80
[40]	*Eucalyptus tereticornis*	Spherical	40–60
[40]	*Melaleuca nesophila*	Spherical	40–60
[40]	*Rosmarinus officinalis*	Aggregates like grapes	–
[19]	*Lawsonia inermis*	Distorted hexagonal-like appearance	21
[19]	*Gardenia jasminoides*	Shattered rock-like	32
[41]	*Amaranthus dubius*	Spherical	43 to 220
[42]	*Kappaphycus alvarezii*	Spherical	14.7
[43]	*Padina pavonica*	–	10–19.5
[43]	*Sargassum acinarium*	–	21.6–27.4

Fe_2_(SO_4_)_3_, FeSO_4_·H_2_O, and FeSO_4_·7H_2_O are used as herbicides, micronutrient for crops, an electrolyte in dry batteries, a supplement in animal feed, and as a galvanizer. These salts are also used in the purification of water, sewage treatment, and in textiles [50]. Iron nanoparticles, in the absence of air, act as better antimicrobial agent than in the presence of oxygen. It is because rusting of iron occurs in the presence of oxygen and water.

$$ 2\mathrm{F}\mathrm{e} + {\mathrm{H}}_2\mathrm{O} + {\mathrm{O}}_{2\ }\ \to\ \mathrm{F}{\mathrm{e}}_2{\mathrm{O}}_3 + 2{\mathrm{H}}^{+} $$

Although conflicting reports with reference to toxicity of iron oxide nanoparticles have been received, even then, they are useful in trace amounts [51–56]. However, the experimental evidences gathered thus far indicate that superparamagnetic iron oxide nanoparticles coated with R–COOH or R–NH_2_ are less toxic than bare nanoparticles [57, 58]. For maximum performance, nanoparticles must be of uniform size and there should not be much variation in temperature because the magnetic moment varies with temperature due to the alignment of spins of free electrons [59].

The application of magnetic nanoparticles is not restricted to only material science but has expanded its tentacles in almost all areas of science such as agriculture, biomedical, and engineering [60–62]. The shape and size of these nanoparticles can be controlled if pH, temperature, and concentration of all components, in the presence of a surfactant, are monitored [63–66]. Their properties vary with their dimensions [67, 68]. The nanoparticles should be of different sizes for different uses. For instance, in case of biomedical application, the iron and iron oxide nanoparticles should exhibit superparamagnetic behavior at ambient temperature [69–71] but for their use as therapeutic agent and in diagnosis, they should be uniformly smooth and stable at physiological pH [72]. Fe_2_O_3_ and Fe_3_O_4_ are commonly employed for biomedical application. The degree of structural variation depends on the process of synthesis of the nanoparticles. The superparamagnetism of nanoparticles exists even in the absence of external magnetic field. Iron and iron oxide nanoparticles are used in the removal of organic substances in aqueous medium [73–75].

Although iron and iron oxide nanoparticles are extensively used in MRI, immunoassay, drug delivery system, catalysis, and magnetic material in biology and medicine, their application in today’s life is more significant [76]. The surfaces of nanoparticles used in drug delivery are generally functionalized with drugs, protein, and genetic materials [77, 78]. Since these nanoparticles have increased surface area, they reduce the quantity of the drug to minimum and also reduce the adverse effect of the drug on normal cells [79–82].

Besides their technological applications, iron and magnetic iron oxide nanoparticles are of great fundamental scientific interest. In recent years, emphasis has been given to target drug delivery by superparamagnetic iron oxide nanoparticles [83, 84] and in vivo application such as detoxification and hyperthermia. In order to reduce the toxicity of these nanoparticles, they are generally coated with nontoxic and biocompatible materials. These nanoparticles can bind with drugs, enzymes, and antibodies and subsequently directed to a specific organ or tissue through an external magnetic field [85]. Magnetization of nanoparticle is therefore essential. It is of prime importance that the nanoparticles should be selected from among the transition metal ions which are highly magnetic in nature. Nonfunctionalized iron oxide nanoparticles have been used for labeling leucocytes, lymphocytes, etc. [86–88]. Cellular uptake of iron oxide nanoparticles can be increased by coating them with dendrimers [89]. Magnetic nanoparticle conjugate is made of iron oxide nanoparticles, covalently bind with methotrexate, and can act both as a contrast agent in MRI and drug carrier.

An attempt has been made to review the biogenic fabrication of magnetic iron and iron oxide nanoparticles and their application in drug delivery and cancer therapy and as a sensor for the detection of pesticides.

### Biogenic Fabrication of Magnetic Nanoparticles

Fabrication of iron oxide nanoparticles at room temperature, using tea (*C. sinensis*) polyphenols has been reported [21]. These nanoparticles showed highest rate of bromothymol blue degradation in comparison to Fe-ethylenediaminetetraacetic acid (Fe-EDTA) and Fe-ethylenediamine-disuccinic acid (Fe-EDDS). In another study, Muthukumar and Matheswaran [90] obtained iron oxide nanoparticles using *Amaranthus spinosus* leaf aqueous extracts. These nanoparticles were spherical with rhombohedral phase structure, smaller in size with large surface, and less aggregation than those produced with sodium borohydride. In this, the photocatalytic and antioxidant activities of the leaf extract as well as the sodium borohydride-mediated iron oxide nanoparticles were also studied. *A. spinosus* leaf extract-mediated iron oxide nanoparticles exhibited better photocatalytic and antioxidant activities than those produced by sodium borohydride. Iron nanoparticles synthesized using green tea extracts have been shown to act as Fenton-like catalysts for the degradation of cationic dyes such as methylene blue and anionic dyes like methyl orange [23]. It has been found that iron nanoparticles synthesized from green tea extract removed almost 100 % of methylene blue and methyl orange at an initial dye concentration of 10 and 100 mg L^−1^. However, when iron nanoparticles were synthesized using the conventional borohydride reduction method, the efficiency was somewhat less for methylene blue (96.3 % for 10 mg L^−1^ and 86.6 % for 100 mg L^−1^) and significantly less in the case of methyl orange (61.6 % for 10 mg L^−1^ and 47.1 % for 100 mg L^−1^). Fe_3_O_4_ nanoparticles were synthesized by hydrothermal method using aloe vera plant extract [91]. Authors have reported that with increase in the reaction temperature and time resulted in magnetite nanoparticles with increased crystallinity and saturated magnetization. Herrera-Becerra et al. [92] have synthesized iron oxide nanoparticles by exposing pretreated and milled powder of *Medicago sativa* to the salt solution of ferrous ammonium sulfate. For this fabrication, 48 h was given. At pH 10, smaller particles with greater proportion of Fe_2_O_3_ were produced whereas larger nanoparticles were produced at lower pH (pH 5).

**Fig. 1:**
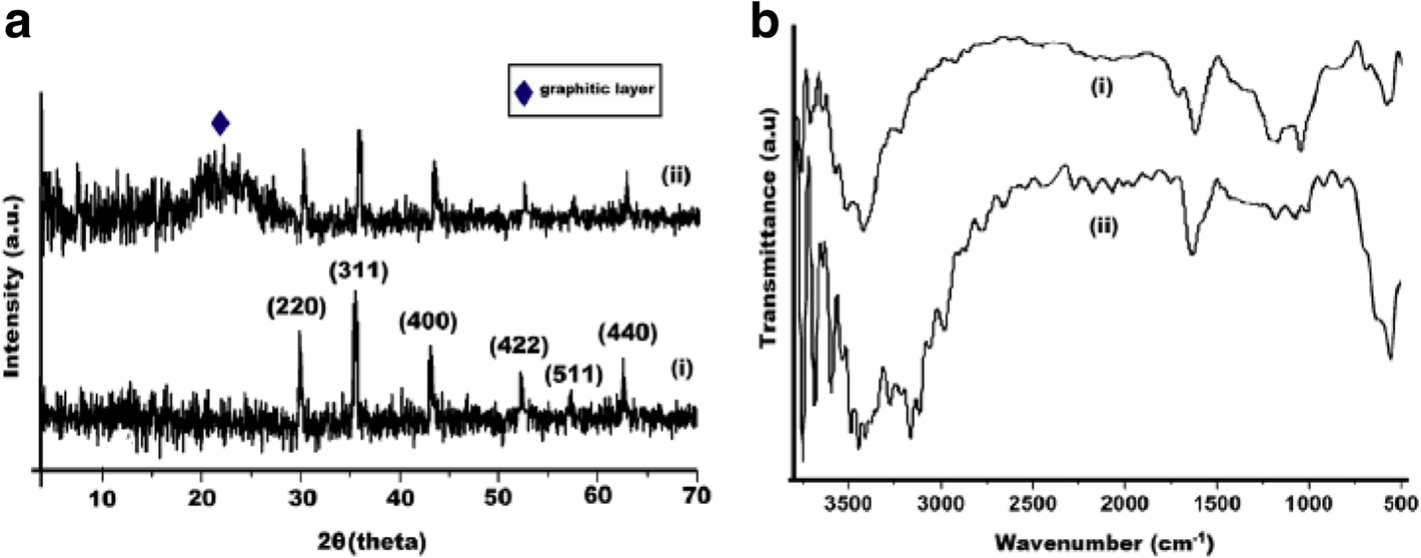
**a** XRD patterns of (*i*) iron oxide nanoparticles and (*ii*) iron oxide/reduced graphene oxide nanohybrid and **b** FTIR spectra of (*i*) reduced graphene oxide nanohybrid [94] and (*ii*) iron oxide/reduced graphene oxide nanohybrid [32]

Lunge et al. [93] have synthesized magnetic iron oxide nanoparticles of 2–25 nm with cuboid/pyramid structure using tea waste template. They exhibited high adsorption capacity for arsenic. It showed very low cost (Rs. 136 per kg). These nanoparticles may be reused up to 5 cycles and regenerated using NaOH. The estimated cost of As(III) removal from water was estimated to be negligible. Leaf extracts of 26 plants were used for the production of nanoscale zero-valent iron particles [37]. The optimum temperature (80 °C) was noted; however, for the extraction time and leaf mass, solvent volume ratio was varied according to the leaf type. Thakur and Karak [32] used banana peel ash extract to synthesize iron oxide nanoparticles; and aqueous extract of *Colocasia esculenta* leaves was used to reduce graphene oxide. Iron oxide formation was validated by XRD (peaks at 30.15, 36.2, 43.32, 53.89, and 29) and FTIR (stretching Fe–O) (Fig. 1a, b). In this study, the nanohybrids exhibited a good reusability with insignificant decrease in efficiency even after the third cycle [32].

**Fig. 2:**
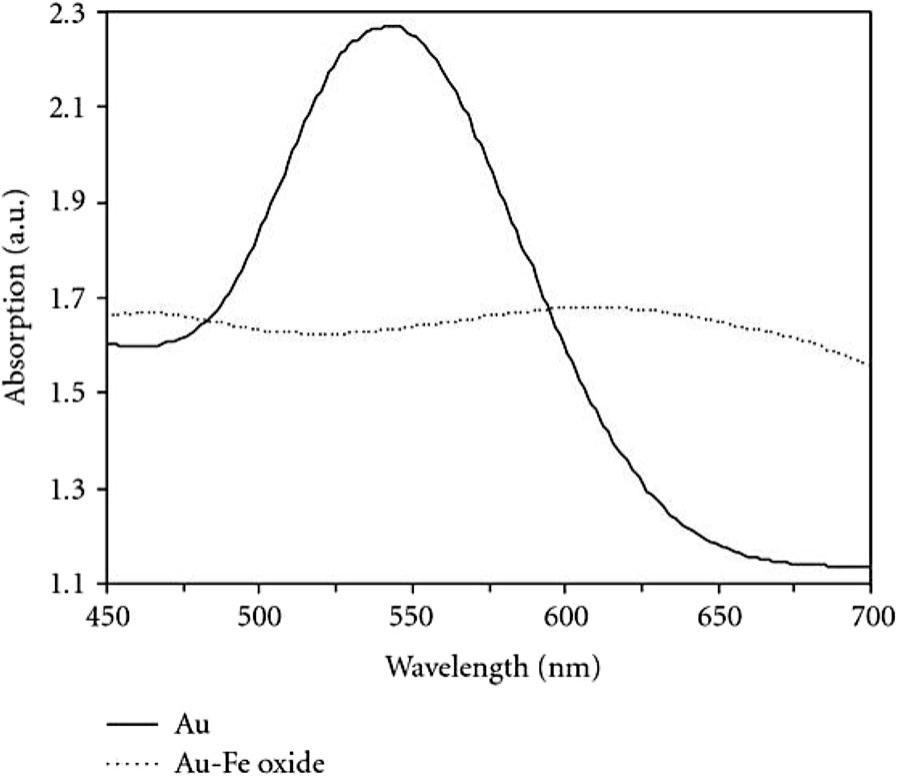
Absorbance spectra of gold nanoparticles (*solid line*) and magnetite-gold composite nanoparticles (*dotted line*) [95]

Au–Fe_3_O_4_ composite with magnetic core was primarily produced by co-precipitation of Fe^2+^ and Fe^3+^. Further, *Eucalyptus camaldulensis* was used for the reduction of Au^+3^ on the surface of magnetite nanoparticles and for the functionalization of the Au–Fe_3_O_4_ nanocomposite particles [95]. UV–vis spectra showed a redshift due to the surface plasmon resonance of Au. The highest absorbance was observed for gold nanoparticles at 530 nm (solid line curve) whereas Au– composite nanoparticles showed a peak at 608 nm (dotted line) which agreed with previous reports (Fig. 2) [96–99]. It has also been observed by many other researchers [96–98, 100].

Senthil and Ramesh [35] synthesized iron oxide nanoparticles by the reduction of ferric chloride solution using *Tridax procumbens* leaf extract containing carbohydrates with an aldehyde group as a reducing agent. Further, these nanoparticles exhibited antibacterial activity against *Pseudomonas aeruginosa*.

Recently, Ehrampoush et al. [33] have reported that the size of iron oxide nanoparticles synthesized from tangerine peel is dependent on its concentration. The size of nanoparticles decreases with increasing concentration of tangerine peel extract from 2 to 6 %. In addition, they have reported that these nanoparticles can be a good adsorbent for the removal of cadmium from wastewater.

### Biomedical Application of Magnetic Nanoparticles

#### Therapeutic Applications

Hyperthermia is a simple technique used to destroy tumor cells by raising their temperature to 41–45 °C. Since the living cells can undergo repair, they are rejuvenated while damage of the tumor cells is an irreversible process [101–103]. This magnetic hyperthermia is more effective when the iron oxide nanoparticles (IONP) are small and uniform. Their surface is generally made biocompatible by coating them with organic polymers or bioactive molecules for their slow release.

#### Cellular Labeling and Cell Separation

In vivo cell separation can be done by cell labeled with ferro paramagnetic substances [104] as the labeled cells can be detected by MRI [105]. They can be labeled by one of the two techniques: (a) attaching magnetic particles to the cell surface [106] or (b) internalizing biocompatible magnetic particles by fluid phase endocytosis [107]. The most appropriate technique for cell labeling is to modify the nanoparticle surface with a suitable ligand such as transferrin, lactoferrin, albumin, and insulin which are generally biocompatible. Such receptors have been shown [108] to internalize without disturbing the nanoparticles. Gupta and Gupta [66] have demonstrated that supramagnetic nanoparticles derivatized with proteins like lactoferrin, transferrin, and ceruloplasmin have strong affinity for receptors on the human fibroblasts surface, which inhibit the phagocytosis. These nanoparticles of less than 20 nm size have high magnetization value. Their influence on dermal fibroblast has been assessed in terms of adhesion viability and morphology by SEM and TEM images.

The interaction of the protein-coated nanoparticle is size dependent since different particles respond differently. Although there is a significantly visible difference in the interaction between fibroblast cell and coated/uncoated supramagnetic nanoparticle, no attempt has been made to realize the magnetic moment value of iron oxide nanoparticles, which changes as a consequence of its binding with proteins and other biomolecules. Iron in the trivalent state in Fe_2_O_3_ is in high spin state with five unpaired electrons in its d orbital but the moment it is coated with proteins, it goes to low spin state with a consequent change in the repulsion and magnetic moment value from 5.91 BM to 1.73 BM corresponding to one unpaired electron. It is due to the complex formation of Fe^3+^ with protein which being a strong ligand forces the electrons to be paired up. As a result, the Fe^3+^ is reduced in size but surface area increases due to complexation with the protein. The magnetic moment and reduction in size of Fe_2_O_3_ nanoparticle seems to be the key factor in the process of internalization and phagocytosis. It has been suggested that tissue repair can be done either by welding or soldering when polymer-coated nanoparticles are placed between two tissue surfaces. Temperature greater than 50 °C is produced by denaturation of tissue and also by absorption of light by coated nanoparticle [109].

#### Tissue Repair

The above method suggested for tissue repair is not convincing as the same temperature is produced to destroy the tumor cell which is supposed to join the two damaged normal cells [66]. The hypothesis first shows denaturation and then connecting the cells through other proteins. It appears as if the denaturation has twofold purposes; some proteins are disintegrated from the cell at 50 °C and some proteins remain unaffected which subsequently join the cells together. When gold/silica-coated Fe_2_O_3_ nanoparticles are coated on the tissues, they may prevent further damage but it seems unlikely that two unit cells are joined together. However, the self-repairing of the damaged tissue is a natural process that does not require raising the temperature of the tissues.

#### Treatment of Cancer

Wu et al. [109] have shown that gold-coated iron nanoparticles suppress cancer cell growth in oral and colorectal cancer cells in vivo and in vitro [110]. Although the healthy cells are equally exposed to iron nanoparticle, they are not much affected and the replication of cancer cells is inhibited. The cytotoxicity is due to the magnetic properties of the elemental iron nanoparticle; the oxidation of which is delayed by coating them with gold. As the oxidation of iron nanoparticles begins, the cytotoxicity decreases towards cancer cells. In fact, the gold coating slowly dissolves to release the iron nanoparticle. The reactive oxygen species (ROS) is generated which triggers the process of cytotoxicity. It has also been observed that the addition of ROS scavenger does not protect the cancer cells from the nanoparticles with an iron core and gold shell (Fe@Au)-induced cytotoxicity.

**Fig. 3:**
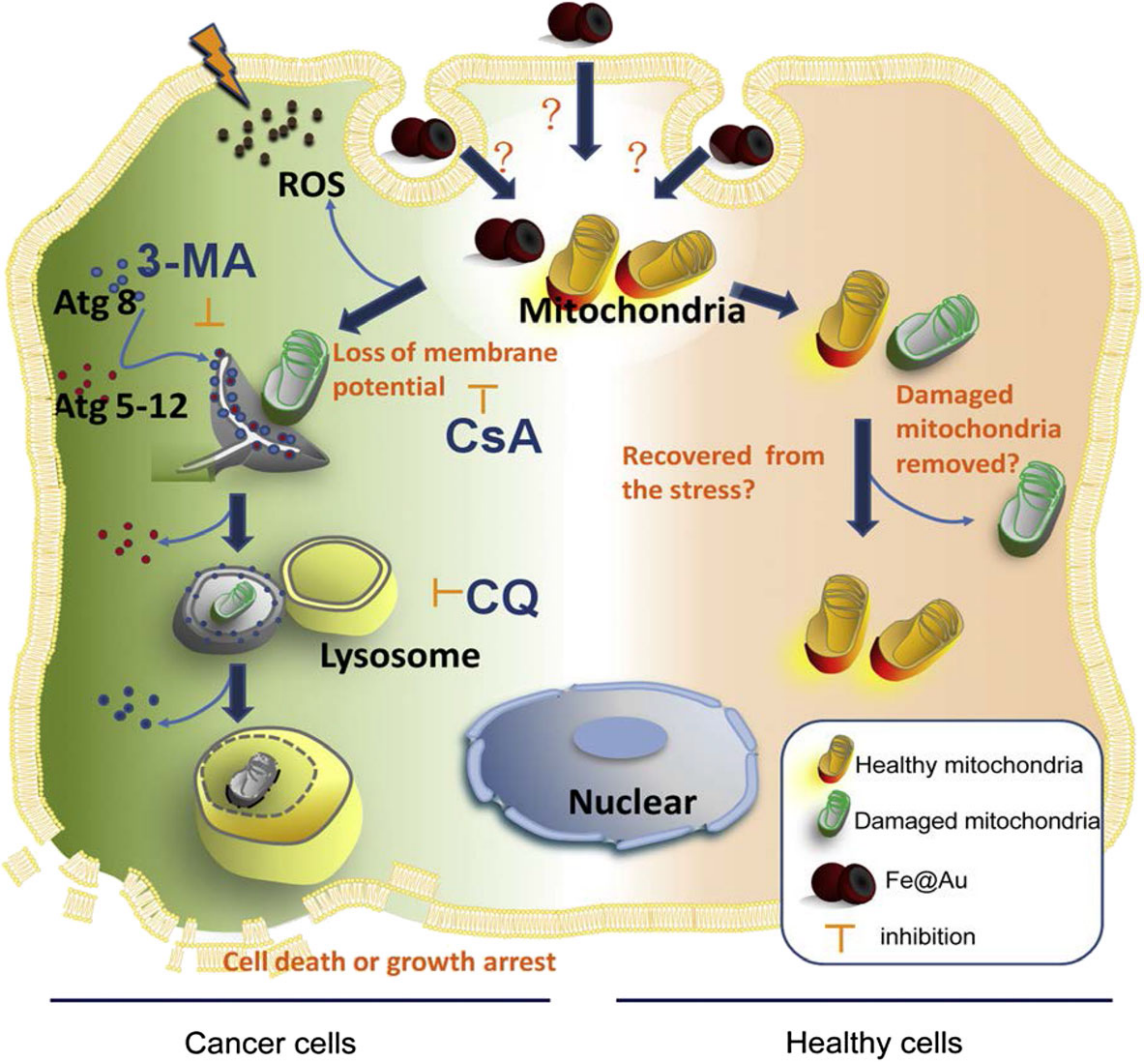
Fe@Au induced a cancer cell-specific cytotoxicity through the mitochondria-mediated autophagy [109]

Decrease in mitochondrial membrane potential in cancerous cells occurs when treated with Fe@Au, although it is not clearly known as to how it interferes with the normal function of the mitochondria. Since the mitochondria are redox sensitive, they are targeted by Fe@Au. Iron is slowly oxidized, due to which, perhaps the mitochondrial membrane potential decreases. The cytotoxicity of Fe@Au towards cancer cells is an irreversible process, while the healthy cells are also affected but they recover within 24 h. The oxidation of iron nanoparticles and generation of ROS are simultaneous processes (Fig. 3).

$$ \mathrm{F}{\mathrm{e}}^0@\mathrm{A}\mathrm{u} + \mathrm{mitochondria}\to\ \mathrm{F}{\mathrm{e}}^{\mathrm{n}+}@\mathrm{A}\mathrm{u}\mathrm{O} + \mathrm{mitochondria} $$

Fe@Au caused a shock to the mitochondria within 4 h, but the cancer cells could not recover from the damage caused by them. Further, it caused a sequential autophagy and inhibited the cancer cell growth.

#### Drug Delivery

**Fig. 4:**
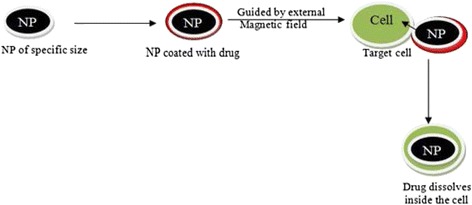
Drug delivery through nanoparticles on the target cells

Mahmoudi et al. [58] have studied the application of supraparamagnetic iron oxide nanoparticle (SPION) in drug delivery. The drugs are bound on SPION surface or encapsulated in magnetic liposomes and microspheres. SPIONs can deliver peptides, DNA, chemotherapeutics, and radioactive and hyperthermic drugs. They are designed such that the drug or ligand is bound to its surface and guided with an external magnetic field to the desired site. The nanoparticles enter the target cells and deliver the drug there (Fig. 4).

When the drug is dissolved inside the target cell, the SPIONs are exposed to other cells. This process can reduce the quantity of drug, absorption time, and interaction of drug with nontarget cells. However, it is essential that iron nanoparticles should be magnetic and smaller than the target cells so that they can easily diffuse into them. Since large quantity of SPIONs cause agglomeration, their high concentration may be avoided. From in vivo experiments, it has been shown that in most cases, the drug is immediately delivered due to the bursting of the nanobubbles carrying the drug, as a consequence of which insufficient quantity of the drug reaches the target cells.

Mahmoudi et al. [111] synthesized cross-linked polyethylene glycol co-fumarate-coated iron oxide nanoparticles and loaded them with tamoxifen to see if it can reduce the burst time. Interestingly, it was found to reduce the burst time by 21 %. In a similar experiment, Guo et al. [112] loaded monodispersed SPION having a mesoporous structure, with doxorubicin, and observed that it had very high drug loading capacity and slow release. The drug release can be controlled by permeability, temperature, sensitivity, pH, surface functionality, and biodegradability of nanoparticles [113].

Colloidal magnetic nanoparticles are used in drug delivery at a desired target without interacting with other living cells. In the case of breast cancer (BT 20 cells), polyethylene glycol (PEG)-coated nanoparticles ranging between 10 and 100 nm were found to penetrate into the cells [114]. It is believed that since PEG is appreciably soluble in both polar and nonpolar solvents, it releases the magnetic nanoparticles at the tumor cells. Gupta and Gupta [66] have shown that the efficacy of magnetic microspheres in the targeted delivery of the incorporated drug is mainly due to magnetic effects. However, it is true for magnetic nanoparticle only, but innocuous and biocompatible nanoparticles of other metals such as gold and silver of smaller size [115] are known to be more effective than the larger particles of the same metal as drug carrier [116]. This technique is cost effective and reduces the quantum of drug to be transported to the site of use. Also, it protects the normal healthy cells from adverse effects of the drug.

Since the magnetic iron oxide nanoparticles are an excellent drug carrier, they are used in chemotherapy. However, iron oxide nanoparticles do not influence the human fibroblasts cells (IMR-90). It means that they are selective towards cancer cells. Khan et al. [117] demonstrated that when both the cancer cells (A549) and normal cells are exposed to iron oxide nanoparticles in a concentration range of 10–100 μg/ml for 24–48 h, necrosis of cancer cells occurs leading to their death. Loss of mitochondrial membrane potential and depletion of ATP suggest that necrosis is the major cause of cytotoxicity of cancer cells rather than apoptosis. ROS generation has been evidenced in A549 cells which are concentration and time dependent.

A variety of bimetallic nanoparticles of the type MFe_2_O_4_ (where M = divalent Mg, Fe, Co, Ni, Cu, and Zn) containing two metal ions has been reported for biomedical applications. Their magnetic properties are dependent on the number of unpaired electrons in the d orbital of transition metal ions. Multifunctional magnetic nanoparticles (MNPs) can be prepared by coating them with gold, silica, zinc oxide, polymer, liposome, etc. They can be further functionalized to make MNPs stable and multifunctional [118]. Xu and Sun [119] have attempted to deliver cisplatin to solid tumor through Fe_3_O_4_ HMNPs.

They have produced small pores in the polycrystalline nanoparticles by heating in oleic acid which allows the drug to be diffused easily in these pores. It has been shown schematically that the drug is delivered via ligand exchange. However, it is known that cisplatin is labile in aqueous medium and can react with water as shown below followed by exchange with surfactant.

Magnetic targeting of diseased cells by SPION has been done in some cases [120]. In order to increase the target yield, SPIONs are generally coated with polymers and functionalized by attaching carboxyl groups, biotin, avidin, carbodiimide, or any other biomolecule [121–123]. When the drug is carried at the target cell (tumor cell), it has to be released either by external force or by changes in pH, osmotic pressure, or temperature [124]. The drug is then picked up by tumor cells and penetrates via diffusion [125]. For such application, it is essential for the SPIONs to be stable at neutral pH and physiological conditions. The stability of the colloidal solution is dependent on the dimension of the nanoparticle and their aggregation may be prevented by coating them with an appropriate substance [126]. Larger particles (larger than 10 nm) cannot penetrate the endothelium [127] under normal conditions, but they can easily penetrate the tumor cells and inflamed cells [128]. When the coated nanoparticles enter the tumor cells, the coating is dissolved in the biological fluid and they are exposed to other cellular components [111, 129].

However, if the concentration is increased, aggregation of nanoparticles may occur leading to greater magnetic interaction. It is also believed that agglomeration of nanoparticles in the capillaries may block their passage [130]. Since the biological pH and the isoelectric point of SPION at pH 7 are the same [131, 132], they influence the colloidal stability of the SPIONs [133, 134].

It is essential that the drug-carrying spherical particles must always be smaller than the RBC and the blood capillaries where the drug is injected. The nanoparticles after drug delivery are exposed to normal cells, and therefore, it is essential that they should be nontoxic to them.

#### Effect of Internalization of Nanoparticles

Calero and others [135] have studied the effect of internalization of magnetic iron oxide nanoparticle on HeLa cells in vitro. They also assessed the damage of normal healthy cells and production of ROS. The internalization was found to be dependent on the type of coating of MNPs and their concentration. It was, however, noticed that besides the increasing concentration of MNPs (0.05, 0.1, and 0.5 mg ml^−1^), the uptake of APS-coated iron oxide nanoparticles by cells was higher than those coated with AF or dimercaptosuccinic acid (DMSA) [136]. The charge and surface of MNPs are important since positively charged particle surface is attracted towards negatively charged surface by default. Calero et al. [135] have observed that APS-coated positively charged nanoparticles are capable of penetrating easily into the HeLa cell than the DMSA- and AD-coated MNPs. It can be understood that positively charged MNPs are smaller than the negatively charged species, and therefore, being smaller in size, they can easily diffuse into the cells which has also been demonstrated by Kenzaoui et al. [137] in a separate experiment. The entrance of MNPs follows endocytosis [138, 139]. Although substantial number of MNPs accumulates in the cytoplasm and does not reach the nucleus, genotoxic damage of iron oxide MNPs due to the production of ROS occurs, irrespective of the cell type, coating, or their concentration. Such nanosized magnetic materials may therefore be used in medical diagnosis, especially in the identification of cancer cells, MRI, and carrier for drug delivery.

#### Tumor Treatment

It was observed from a study of cancer cells exposed to iron oxide nanoparticles that the cell death occurs by necrosis rather than apoptosis. Interestingly, it was also found that when normal human fibroblasts cells were exposed to iron oxide nanoparticles, insignificant cell death occurred. It demonstrates that these nanoparticles can be safely used in the treatment of tumors without damaging the healthy cells.

The ROS generation in this system was examined by the probe when cancer cells A549 were treated with iron oxide nanoparticles. The maximum ROS was generated after 24 h at a rate of 100 μg/ml iron oxide nanoparticles which subsequently induced autophagy [117].

#### Antibacterial Activity

Effect of iron nanoparticles on the deactivation of *Escherichia coli* has been studied by Lee et al. [140] under aerobic and anaerobic conditions. It was observed that in the absence of oxygen, the inactivation of *E. coli* was at maximum when exposed to 9 mg L^−1^ of iron nanoparticles for 10 min.

In air-saturated solution containing as high as 90 mg L^−1^, iron nanoparticles in *E. coli* solution exposed for 90 min had negligible inactivation of the bacteria. This is particularly due to the presence of oxygen which oxidizes the Fe^0^→Fe^2+^ and also the absence of hydroxyl radical. The iron nanoparticles are oxidized to FeO and Fe_2_O_3_ which form a film on the surface of the nanoparticles preventing the lower layer from further corrosion [141].

$$ \mathrm{F}{\mathrm{e}}^0\ \to \kern0.5em \mathrm{F}\mathrm{e}\mathrm{O}\ \to\ {\mathrm{F}}_2{\mathrm{O}}_3 $$

However, when a chelating agent such as PO_4_^3−^ ion is added in an air saturated system, the biocidal activity of iron nanoparticle is reduced because Fe(III) forms an insoluble metal chelate with PO_4_^3−^ ion. On the contrary, when oxalate ion, C_2_O_4_^2−^, is added, the bactericidal activity is enhanced because it forms a soluble complex with the iron ion. This is also evidenced and monitored from a change in color from black to yellow.

$$ \mathrm{F}{\mathrm{e}}^0\ \mathrm{nanoparticles}\ \left(\mathrm{black}\right)\kern0.5em \to\ \mathrm{F}{\mathrm{e}}^{3+}\left(\mathrm{yellow}\right) $$

Since Fe(II) is highly susceptible to oxidation by air, it does not stay stable unless stabilized by an acid.

**Fig. 5:**
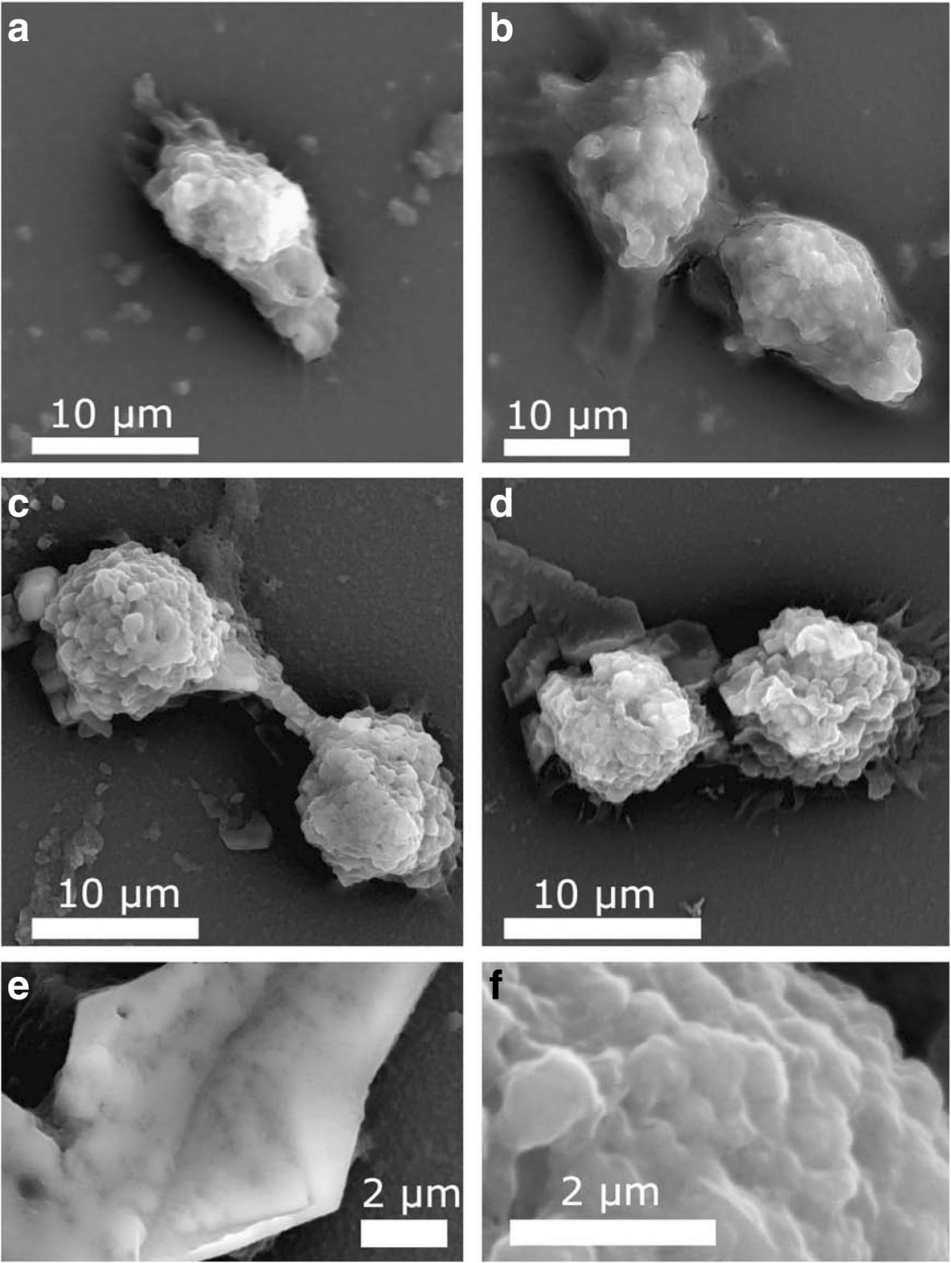
SEM results for **a** control L929 cells and the cells interacted with **b** nanobeads, **c** nanoworms, and **d** nanospheres. Panels **e** and **f** illustrate the higher-magnification image of the surface of control L929 and the one interacted with nanospheres, respectively [156]

Superparamagnetic iron oxide nanoparticles are frequently used as magnetic drug targeting, MRI, tissue repair, etc. [142–146]. They are useful in drug delivery. Since they can be guided by external electric field to the desired target, they can stay there when the magnetic field is cut off. Fe_3_O_4_ can be synthesized by a variety of procedures. Their size and shape may be controlled by monitoring the pH, temperature, and concentration of the reacting components. Coating with a suitable substance can prevent their agglomeration. SPIONs (800 nm) were introduced to the fibroblast cells (Fig. 5) to examine the changes in their morphology [147].

**Fig. 6:**
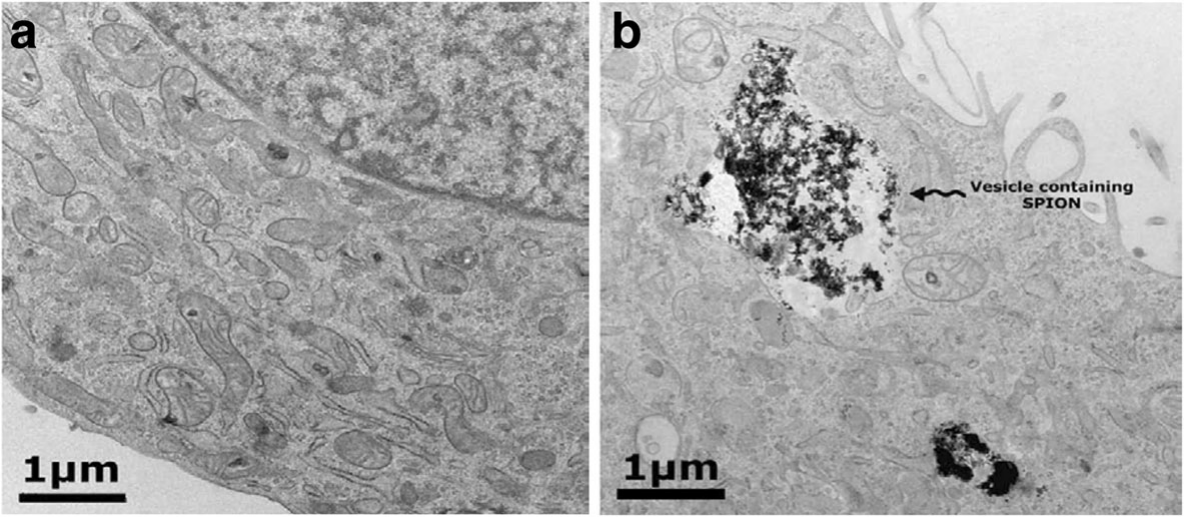
TEM images of L929 cells for **a** control and **b** cells exposed to SPION with vesicle-containing SPION nanospheres [156]

It was found that the toxicity increases with the shape of SPIONs such as nanobeads, nanowires, and nanospheres. The deformation of the cell increases with the concentration of SPIONs. The TEM images of fibroblast cells exposed to SPION showed that smaller nanoparticles penetrate into the cell (Fig. 6) while larger ones have not been traced into them. The size is, therefore, of prime importance in such cases, although the coating also influences the size of the nanoparticles.

Although efforts are being made to produce SPION coated with organic molecules for use in human system, only dextran-coated SPIONs have so far been approved by Food and Drug Administration (FDA) [135].

Mahmoudi et al. [49] have evaluated the toxicity of bare SPION and those coated with –COOH and –NH_2_ against human cell lines HCM (heart), BE-2C (brain), and 293T (kidney). The toxicity of bare SPION was found to be higher than those coated with organic molecules due to their greater affinity to absorb proteins, vitamins, amino acids, and ions causing change in pH of the medicine [148–150]. Since the human cell contains proteins, vitamins, and amino acids, they have affinity to bind with SPION whereas the SPIONs already coated with these groups do not have vacant space for chelation with them. The toxicity of the coated SPIONs is, therefore, less than those of uncoated ones. The low toxicity of coated SPION is useful in the detection of cancer cells because they do not damage the normal cells. One type of SPION is toxic to certain types of cells while the other types produce insignificant effect. For instance, Mahmoudi and others [49] have reported that negative SPIONs did not produce significant changes on the cytoskeleton of heart cells compared to high toxic effect on the cytoskeleton of both kidney and brain cells.

### Environmental Application of Magnetic Nanoparticles

#### Removal of Dyes

The iron nanoparticles produced from green tea leaf extract have been shown to contain iron oxide and oxohydroxide [23]. They have been used as a Fenton-like catalyst for the removal of organic dyes such as methylene blue and methyl orange in aqueous medium. It has been shown that they are highly effective in the removal of cationic and anionic dyes over a wide range of concentrations (10–200 mg L^−1^). Also, the iron nanoparticles synthesized from green tea leaf extract are more effective than those produced from borohydride reduction. Shahwan et al. [23] have reported that the iron nanoparticles were washed with ethyl alcohol to remove NaCl from the sample. Since NaCl is an ionic salt, it is soluble only in water and cannot be removed by washing with ethyl alcohol. It was also noted that the pH of methylene blue and that of methyl orange containing iron nanoparticles was around 8.30. However, the pH started declining immediately after the addition of H_2_O_2_ until it became constant at 3.11 after about 6 h when the dye was completely removed.

#### Pesticide Detection Sensor

Because of their application in agriculture, pesticides have become one of the major environmental pollutants. Iron oxide nanoparticle (Fe_3_O_4_) being chemically and biologically neutral have been coated with catalysts, enzymes, or even antibodies to be used as biosensors [151]. In a recently published paper, Chauhan et al. [152] have modified Fe_3_O_4_ nanoparticle using poly(indole-5-carboxylicacid) by preparing nanobiocomposite for its use as a sensor for the determination of pesticides such as malathion and chlorpyrifos in a wide range of concentrations (0.1–70 nm).

Iron nanoparticles in the elemental state have also been used in the purification of ground water. It has been tested against reductive dehalogenation of organochlorine pesticides and insecticides [153–155] and several other toxic substances such as chromium [156] and arsenic [157]. Since agglomeration of iron nanoparticle occurs, it was modified using surfactants. This not only reduces the organochlorine pesticides but also prevents corrosion. Mukherjee et al. [158] have shown that after accepting H^+^, the hepatochlor pH increases which is true but the term reduction used by them is incorrect as the acceptance of proton, by definition, is oxidation.

Pesticides used in agriculture are sometime harmful to other animals and plants. Their reduction to innocuous chemicals by iron nanoparticle is a simple strategy to make them useful. Polyhalogenated and nitroaromatic compounds are generally reduced by metal nanoparticles, metal sulfides, quinine, and vitamin B_12_. Metal nanoparticles can also be used for the reduction of nonhalogenated pesticides and azo dyes. The findings made by Keum and Li [159] suggest remediation, but it is too expensive to be used in the field. Also, it leaves toxic residues in the environment.

## Conclusions

Iron and iron oxide magnetic nanoparticles can be fabricated by plant extracts or microbes such as fungi and microalgae. They can be coated with water soluble polymers for greater solubility. For instance, polyvinyl alcohol-coated nanoparticles are prevented from aggregation; therefore, they can easily diffuse through the semi-permeable membrane in a living system. Their shape and size may be controlled by maintaining the temperature, pH, and concentration of the reacting components. Their cytotoxicity varies with shape such as spherical, beads, and rods. The hydrodynamic size and the paramagnetic/diamagnetic nature of iron oxide nanoparticles make them specific for specific application. For example, nanoparticles with larger hydrodynamic size have lower cytotoxicity. Superparamagnetic iron oxide nanoparticles have great potential for use in instruments and medical devices, as drug carrier, and in the treatment of many diseases. Microbes and plant extracts containing alkaloids, flavonoids, saponins, ketones, aldehydes and phenols, or reducing acids like citric acid and ascorbic acids can be used for nanoparticle synthesis. Iron oxide nanoparticles can be used as an inexpensive material for the removal of dyes from textile industries and tanneries and in the treatment of contamination in wastewater and purification of ground water. Since the properties of iron oxide nanoparticles solely depend on the number of unpaired electrons and particle size in the presence and absence of magnetic field, it can be altered by coating them with polymers and by applying external magnetic field. These nanoparticles can be selectively used for the separation of magnetic materials from a huge deposit of nonmagnetic substances.
